# Non-Invasive Ventilation with Neurally Adjusted Ventilatory Assist (NAVA) Improves Extubation Outcomes in Extremely Low-Birth-Weight Infants

**DOI:** 10.3390/children11101184

**Published:** 2024-09-28

**Authors:** Kevin Louie, Shaili Amatya, Gad Alpan, Lance A. Parton

**Affiliations:** 1 Division of Newborn Medicine, Maria Fareri Children’s Hospital, Westchester Medical Center and New York Medical Center, Valhalla, NY 10595, USA; kevin.louie@vumc.org (K.L.); samatya@pennstatehealth.psu.edu (S.A.); gadialpan@gmail.com (G.A.); 2 Department of Pediatrics, Division of Neonatology, Vanderbilt University Medical Center, Nashville, TN 37232, USA; 3 Division of Neonatal-Perinatal Medicine, Department of Pediatrics, Penn State Children’s Hospital, Hershey, PA 17033, USA

**Keywords:** neurally adjusted ventilatory assist, non-invasive positive-pressure ventilation, bronchopulmonary dysplasia, extremely low birth weight

## Abstract

**Objective:** This study investigates the effectiveness of extubation from conventional mechanical ventilation using an endotracheal tube (MVET) compared to synchronized non-invasive positive-pressure ventilation (sNIPPV) using neurally adjusted ventilatory assist (NAVA) and conventional non-invasive positive-pressure ventilation (NIPPV) in extremely low-birth-weight (ELBW) infants. **Methods:** An institutional review board (IRB) approved this study (#12175) to conduct a single-center randomized control trial including 60 ELBW infants assigned in a one-to-one computer-generated scheme to either sNIPPV using NAVA or NIPPV. The primary outcome involved the need for reintubation, and the secondary outcome involved the assessment of moderate/severe BPD, defined as an oxygen requirement at 36 weeks, as in #NCT03613987 (clinicaltrials.gov). **Results:** There were 60 ELBW infants enrolled and randomized. The overall gestational age was 26 (1.5) weeks, and the birth weight was 773 (157) g [mean (SD)]. There were no statistically significant differences between the NAVA and NIPPV patient characteristics. There was a 41% extubation failure rate in the NIPPV group and 35% in the NAVA group (*p* = NS). The NAVA group had less moderate and severe BPD (*p* = 0.03), a shorter oxygen therapy duration (*p* = 0.002), a decreased length of stay (*p* = 0.03), and less need for home oxygen (0, 43%; *p* = 0.0004). **Conclusions:** This study found similar extubation failure rates among ELBW infants as in prior studies. However, the NAVA group had lower rates of moderate/severe BPD and need for oxygen at discharge, as well as shorter oxygen therapy duration and length of stay. The use of NAVA may be a reasonable alternative mode of non-invasive ventilation in the ELBW population.

## 1. Introduction

Infants born weighing less than 1000 g at birth (ELBW) are at a high risk of requiring mechanical ventilation with an endotracheal tube (MVET) and progressing to bronchopulmonary dysplasia (BPD) [1,2]. A surfactant deficiency, immaturity of the lung, its vasculature, and the extracellular matrix, as well as the immaturity of the respiratory control center, all contribute to the need for MVET [3]. Numerous ventilator strategies have been utilized to minimize lung injury, particularly non-invasive ventilation (NIV) modalities following a planned extubation. Continuous positive airway pressure (CPAP) and non-invasive positive-pressure ventilation (NIPPV) are two methods that have been used to decrease extubation failure. Less elective extubation failures have been demonstrated when extubation is transitioned to NIPPV compared to CPAP [4,5]. The underlying mechanism of NIPPV is not entirely understood; however, some have suggested that NIPPV provides intermittent distending pressure above the positive end-expiratory pressure (PEEP), which may assist in maintaining functional residual capacity, improving thoracoabdominal wall synchrony, and reducing apnea of prematurity [6,7]. As a result, NIPPV has been implemented as a mode of NIV following a planned extubation.

NIPPV can be synchronized or non-synchronized to the patient’s intrinsic respiratory rate. There are challenges with both modalities of NIV. During non-synchronized ventilation, a programmed respiratory rate is delivered by the ventilator, irrespective of the patient’s respiratory cycle. For apneic infants, it was believed that delivering a set respiratory rate would “entrain” their breathing. However, this has been shown not to be the case in one study [8]. Additionally, in spontaneously breathing infants, increased gastric distension, barotrauma, and increased work of breathing can follow asynchrony [8].

The challenges involved in synchronized NIPPV (sNIPPV) for ELBW infants include small tidal volumes, rapid respiratory rates, and size variability in the patient–equipment interface. Current methods of synchronization include flow sensors, pneumatic capsules, and catheters to monitor the electrical activity of the diaphragm (Edi). Flow sensors are affected by leaks around the nasal interfaces due to the variability in patient size and equipment. In addition, the small tubing is prone to secretion and water accumulations, leading to inadvertent auto-triggering [9]. Pneumatic capsules have been used to measure abdominal wall movement; however, they currently do not allow measurements of tidal volumes and may be triggered by paradoxical abdominal wall movements [10]. Neurally adjusted ventilatory assist (NAVA) is another approach to synchronization using a specialized catheter not affected by these previous issues.

The specialized catheter in NAVA serves two functions: a feeding tube and a method of synchronizing the ventilator to the patient’s breathing with specialized embedded electrodes within the catheter. The electrodes detect the electrical activity produced by the phrenic nerve controlling the diaphragm. The electrical signal is transmitted to the ventilator, and a breath is delivered in proportion to the intensity of the electrical signal. As the work of breathing increases, the diaphragm contracts more forcefully, and a larger electrical signal is generated. As a result, the ventilator delivers a larger pressure-supported breath. At the end of this breath, the patient’s inherent neural reflex stops inspiration after reaching a given volume/pressure, thus providing rapid feedback to the ventilator, and potentially minimizing barotrauma. Additionally, NAVA can detect central apnea, at which point a preset backup rate and pressure is generated, reducing the likelihood of atelectasis and further reducing the need for reintubation. Based on a study using NAVA and CPAP during apnea, NAVA had fewer apnea times, bradycardias, and desaturations when compared to CPAP (11). This method of synchronization may explain some of the suggested benefits of synchronized NIPPV (NAVA) due to improvements in pulmonary mechanics, decreased work of breathing, and improved extubation success in preterm infants [5,10,11,12,13,14,15].

To our knowledge, there are no randomized controlled trials comparing sNIPPV using NAVA with conventional NIPPV in ELBW infants during elective extubation. We aimed to compare the effectiveness of extubation from conventional MVET to sNIPPV using NAVA with extubation to NIPPV in ELBW infants. We hypothesized that there would be a decreased need for MVET when using sNIPPV compared to NIPPV.

## 2. Materials and Methods

### 2.1. Patient Recruitment and Eligibility

An institutional review board (NYMC IRB) approved this study (#12175). This study was conducted at a single-center, regional level IV NICU. Infants born weighing less than 1 kg at birth, including gestational ages between 24 and 30 weeks, were assessed for eligibility, receiving surfactant within 90 min of birth, and intubated. Mothers were approached for consent in the labor and delivery unit or the NICU after birth. Infants were excluded if a diagnosis of grade 3 or 4 intraventricular hemorrhage (IVH) was known prior to consent; if congenital anomalies, including neuromuscular disorders, were present; or if infants did not require intubation before 7 days of life. This was outlined in #NCT03613987 (clinicaltrials.gov).

### 2.2. Randomization and Intervention

As the standard of care in this NICU, ELBW infants requiring MVET were first placed on conventional mechanical ventilation (CMV) using assist control (AC). If these infants were unable to achieve adequate oxygenation or ventilation, they were escalated to high-frequency oscillatory ventilation (HFOV) or high-frequency jet ventilation (HFJV).

Consented ELBW infants were randomized to sNIPPV using NAVA or NIPPV in a one-to-one computer-generated scheme. Infants randomized to NAVA had an Edi catheter placed. The position was confirmed utilizing the catheter-positioning screen on the Servo-I ventilator (Maquet Critical Care; Solna Sweeden). After the confirmation of placement, assigned infants who met the extubation criteria were extubated to NAVA on the Servo-I ventilator. Infants randomized to the conventional NIPPV group were extubated to the Servo-I or Puritan Bennett 980 ventilator (Medtronic, Minneapolis, MN, USA). Both groups used RAM cannulas as the nasal interfaces.

The NIV management strategies for NAVA and NIPPV were determined by the blood gases, the infant’s clinical status, and ultimately the attending neonatologist. Providers were not blinded due to the need to perform NIV ventilator adjustments.

### 2.3. Extubation Criteria

Infants were extubated following 12 h of clinical stability after achieving the following criteria: mean airway pressure < 8 cm H_2_O, FiO_2_ < 0.4, pH > 7.2, and pCO_2_ < 70.

### 2.4. Reintubation Criteria

Infants were reintubated if they had one apneic event requiring positive-pressure ventilation (PPV); more than 6 apneic events requiring stimulation within a 6 h period; pH less than 7.2; pCO_2_ greater than 70; FiO_2_ greater than 0.6; or if the baby was failing NIV (either strategy) based on the attending neonatologist’s determination.

### 2.5. Outcomes

The primary aim was to assess infant extubation failure on NIPPV and NAVA. Extubation failure was deemed to have occurred if the infant required reintubation within 7 days of extubation and met the reintubation criteria. The infants who were electively reintubated for surgical treatment were not noted as instances of extubation failure. In this study, none of the infants were electively reintubated within 7 days of extubation. As a secondary aim, infants were assessed for moderate and severe BPD as defined by an oxygen requirement of less than 30% or an oxygen requirement greater than 30% supplemental oxygen at 36 weeks PMA, respectively.

Patient and respiratory characteristics were collected. A patient was defined as small for gestational age (SGA) if they had a birthweight lower than the 10th percentile on the Fenton Curve. A moderate/large patent ductus arteriosus (PDA) was defined as requiring either medical or surgical intervention. Sepsis was defined as a positive blood culture.

### 2.6. Data Analysis

The sample size was calculated based on our NICU data (2015–2016) showing that 84% of ELBW infants have a need for MVET at 7 days of life. We hypothesized that sNIPPV using NAVA would decrease the need for MVET at 7 days of life by 40%. For a power of 80% and an alpha of 0.05, the sample size needed would be 27 infants in each group. Anticipating a 10% dropout rate, 30 infants in each arm were targeted.

A *t*-test was used for continuous parametric data; we used a Mann–Whitney Rank-Sum test for continuous non-parametric data, and a Chi-squared or Fisher Exact test for categorical data. Data were summarized using the mean (SD) or median (IQR) for continuous parametric and non-parametric data, respectively. The level of significance was set at 0.05. SPSS version 16 was used for analyzing the data.

## 3. Results

**Figure 1:**
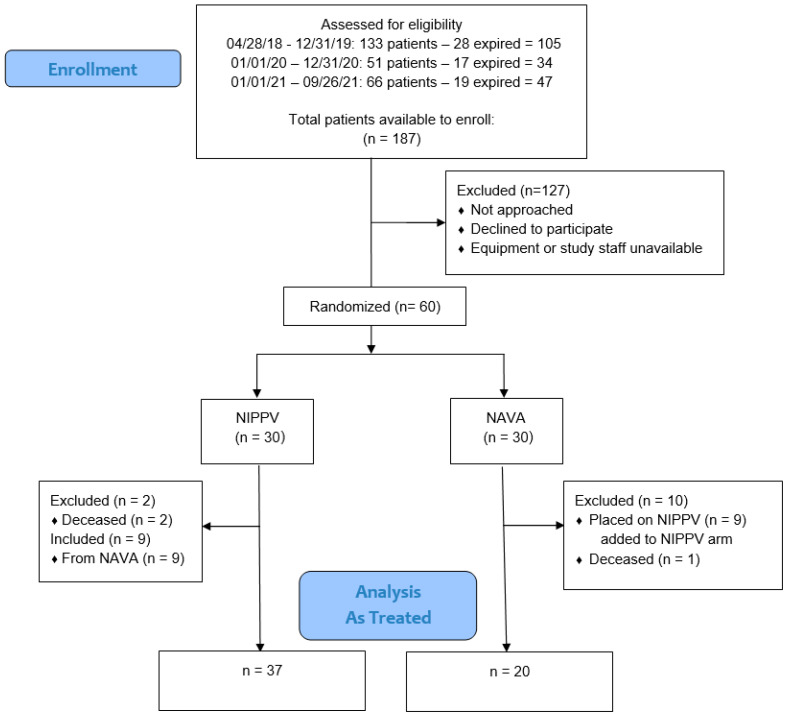
Consort diagram of neonates shown after exclusions and randomization. NIPPV, Non-invasive positive-pressure ventilation; NAVA, neurally adjusted ventilatory assist.

This study was conducted from April 2018 to September 2021. There were 60 infants enrolled, 30 in each interventional arm (See Figure 1). Three infants died: two died prior to extubation, and one infant died two months after extubation for reasons unrelated to respiratory causes. Nine infants, randomized to NAVA, were instead placed on NIPPV due to medical team preference. We analyzed our results using both as-treated and intention-to-treat analyses. No infants received cross-over treatment.

The results for the as-treated groups revealed statistically similar baseline characteristics (Table 1). The mean birthweight was 754 g (155) and 808 (158) (*p* = NS) in the NIPPV and NAVA groups, respectively. The gestational ages were 26 weeks in both groups (*p* = NS). Their respiratory characteristics are summarized in Table 2. All infants received a bolus and the subsequent maintenance of caffeine as standard practices in this NICU prior to extubation. At the time of extubation, the infants in the NIPPV group weighed 1013 g (352) compared to the 870 g (137) weight in the NAVA group (*p* = 0.03). The NIPPV group was extubated at 18 days compared to 3 days of life in the NAVA group (*p* = NS). This absolute difference is attributable to the four (11%) outliers and a somewhat greater skew to the right in the NIPPV group. The NIPPV group also received more diuretics than the NAVA group [24%, 0%; respectively (*p* = 0.02)].

**Figure 2:**
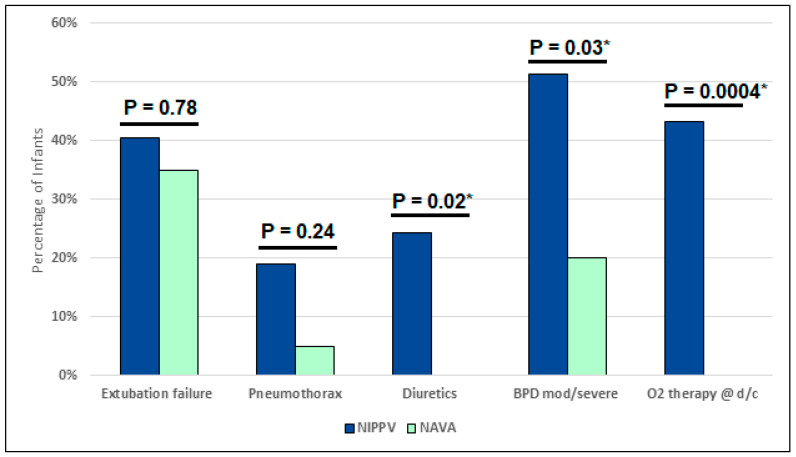
As-treated outcomes comparing mode of non-invasive ventilation. Figure 2 compares the proportion of infants in the NIPPV and NAVA groups in various outcomes such as the incidence of extubation failure, pneumothorax, diuretics use, moderate and severe BPD, and oxygen therapy at discharge. (* *p* = <0.05. NIPPV, non-invasive positive-pressure ventilation; NAVA, neurally adjusted ventilatory assist; BPD bronchopulmonary dysplasia; D/C, discharge).

**Figure 3:**
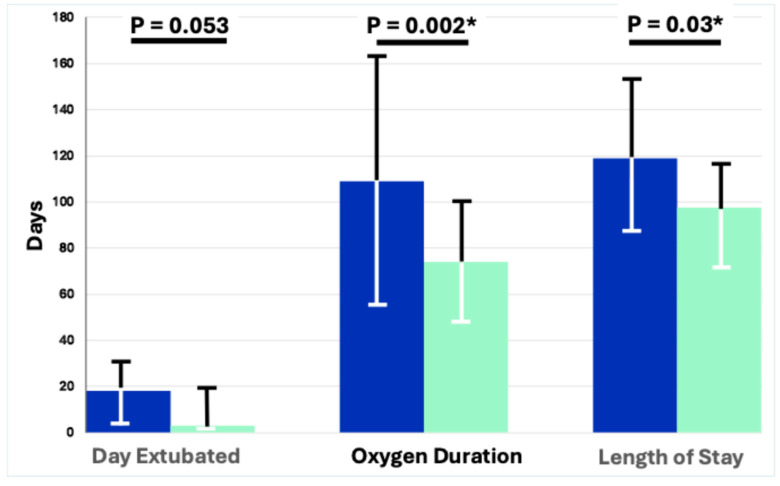
As-treated outcomes comparing modes of non-invasive ventilation. Figure 3 compares the number of infants in the NIPPV to the NAVA group in various outcomes: the day of life when an infant was extubated, the duration of oxygen therapy, and the length of hospital stay in the NICU. (* *p* = <0.05. DOL, day of life; NICU, neonatal intensive care unit; blue for NIPPV; green for NAVA).

Table 3 as well as Figure 1 and Figure 2 represent the primary and secondary outcomes of the as-treated analysis. All pneumothoraces occurred prior to extubation and not while on NIV support. In the NIPPV group, 41% failed extubation compared to 35% in the NAVA group (*p* = 0.78). In both groups, those who failed extubation were reintubated at a median of two days (*p* = NS) post-extubation. The secondary outcomes showed less moderate and severe BPD in those treated with NAVA (20%) compared to those on NIPPV (51%) (*p* = 0.03). There were fewer days on oxygen for the NAVA group—74 days compared to 109 days (*p* = 0.002); no infants on NAVA were discharged home on oxygen versus 43% in the NIPPV group (*p* = 0.0004) (Figure 3). The infants receiving NAVA had a shorter hospital length of stay of 98 days compared to 119 days in the NIPPV group (*p* = 0.03) (Figure 3).

We conducted an intention-to-treat analysis. As with the as-treated groups, this analysis showed no statistical differences in patient or respiratory characteristics (Table 4 and Table 5). The NIPPV group had an extubation failure rate of 43% compared to 34% in the NAVA group (*p* = NS). The rates of moderate and severe BPD in the NIPPV group compared to the NAVA group were 50 and 31% (*p* = 0.18).

## 4. Discussion

The ventilation of ELBW infants continues to be challenging. The ELBW infant has an increased risk from MVET due to multiple factors. There are increased risks of damage to the vocal cords, subglottic area, and infections associated with MVET. Even after elective extubation, it is estimated that up to two-thirds of ELBW infants will require a reintubation at least once during their hospitalization [16]. Additionally, in a large study with over 3000 ELBW infants, BPD rates increased after having 4 or more intubations [17]. This has prompted many providers to trial alternative NIV strategies to prevent intubations and subsequent reintubations. The optimal non-invasive ventilation strategy in the ELBW infant has yet to be determined.

Furthermore, the American Academy of Pediatrics (AAP) clinical report [18] established that synchronized NIPPV effectively reduces the incidence of extubation failure in preterm infants. Other studies, such as Ramos-Navarro et al. [19], used synchronized NIPPV based on a pneumotachograph (flow sensor) to demonstrate that intubation was avoided significantly in preterm infants. Such flow sensors are less affected by leaks [20].

Alternately, NAVA is an ideal synchronization system that detects the neural activity of the diaphragm. NAVA can avoid the unwanted effects of other trigger systems in ELBW patients, such as reliance on abdominal wall movement and flow sensors subjected to leaks. The proposed benefits of NAVA are to enable infants to better control their breathing rate and to have more effort-matched inspiratory times and pressures. This is accomplished through electrodes at the end of a specialized catheter, which may be problematic in ELBW infants with a narrower esophageal width for capturing this signal. The use of NAVA has been studied in larger birthweight infants but this remains to be evaluated in ELBW infants.

This study demonstrated decreased moderate and severe BPD rates, oxygen therapy durations, the need for oxygen at the time of discharge, and lengths of stay in the NAVA group. We speculate that NIV–NAVA offers improved support over NIPPV for ELBW infants by improving respiratory synchrony, resulting in decreased laryngeal ‘braking’. An animal study with newborn lambs has shown that the activation of thyroarytenoid muscles and glottal narrowing with high levels of pressure support the diversion of air away from the lungs and into the esophagus. This response was not seen with NAVA in their study [21]. In our study, this improved ventilation efficiency and decreased the insufflation of the gastrointestinal tract may have facilitated a more rapid weaning from non-invasive ventilation, which may have translated to fewer supplemental oxygen days. While we did not collect inspiratory pressure data, prior studies utilizing NAVA have demonstrated lower peak inspiratory pressure requirements while maintaining the same respiratory parameters compared to NIPPV [15,22]. Lower inspiratory pressures had lung-protective effects in preventing moderate and severe BPD, as seen in our study, resulting in a reduced need for oxygen at discharge. We also found decreased lengths of stay in the NAVA group, possibly related to the aforementioned results, which may also have economic advantages not measured in this study.

The issues of our study may have developed when NAVA was first introduced to our NICU at the start of this study. Training was required for all staff; however, training was not always feasible due to staff availability, new hiring, and the COVID-19 epidemic. This may have contributed to selection bias as infants randomized to NAVA were placed onto NIPPV due to a lack of familiarity with the equipment. Our original power analysis calculation required 30 infants for each group. To mitigate the selection bias, we conducted an as-treated and an intention-to-treat analysis (Table 6), and the results of both analyses were similar.

We also acknowledge that there was no established NIV management strategy. While several primary staff became familiar with NAVA, there were no established weaning strategies regarding NAVA or NIPPV. This provider bias may have affected the secondary outcomes.

Our study was also limited by the lack of data collection on peak inspiratory pressure (PIP) and maximum FiO_2_ during invasive ventilation, which may have influenced the development of moderate and severe BPD.

## 5. Conclusions

In conclusion, we found NAVA to be associated with less moderate and severe BPD, oxygen therapy days, and less need for oxygen at discharge, resulting in a decreased length of stay. While there are limitations to this study, the data suggest that there may be significant benefits in using NAVA post-extubation in ELBW infants. Given the limitations of our study, further randomized trials to confirm these results are needed.

## Figures and Tables

**Table 1:** Patient characteristics compared to each mode of non-invasive ventilation.

	As Treated		*p*-Value
	NIPPV (N = 37)	NAVA (N = 20)	
**Birth weight, g ^†^**	754 (155)	808 (158)	0.22
**Gestational age, wks ^†^**	26 (1.6)	26 (1.2)	0.83
**C/S**	29 (78%)	15 (75%)	0.75
**Male**	18 (49%)	9 (45%)	1
**SGA**	7 (19%)	2 (10%)	0.47
**APGAR 1 min ≤ 7**	36 (97%)	19 (95%)	1
**AGPAR 5 min ≤ 7**	31 (84%)	15 (75%)	0.49
**APGAR 10 min ≤ 7**	17 (46%)	5 (25%)	0.68
**Antenatal steroids**	33 (89%)	18 (90%)	1
**PDA**	28 (76%)	13 (65%)	0.54
**Sepsis**	6 (16%)	1 (5%)	0.40
**IVH grade 3/4**	2 (5%)	3 (15%)	0.22

NIPPV, non-invasive positive-pressure ventilation; NAVA, neurally adjusted ventilatory assist; C/S, caesarean section; SGA, small for gestational age; PDA, patent ductus arteriosus; IVH, intraventricular hemorrhage. ^†^ mean (SD); otherwise listed as N (%).

**Table 2:** As-treated respiratory characteristics.

	As Treated		*p*-Value
	NIPPV (N = 37)	NAVA (N = 20)	
**Surfactant**	37 (100%)	20 (100%)	1
**Received > 1 dose surfactant**	13 (35%)	2 (10%)	0.06
**CMV days pre-extubation ^‡^**	8 (2, 14)	2 (1, 7)	0.09
**HFOV days pre-Extubation ^‡^**	4 (0, 17)	2 (0, 7)	0.22
**HFJV days pre-extubation ^‡^**	0 (0, 3)	0 (0, 0)	0.13
**DOL extubated ^‡^**	18 (2, 34)	3 (1, 20)	0.053
**Wt. at extubation g ^†^**	1013 (352)	870 (137)	0.03 *
**MVET total days ^‡^**	22 (10, 48)	12 (4, 27)	0.07
**Postnatal steroids**	14 (38%)	3 (15%)	0.13
**Diuretics**	9 (24%)	0 (0%)	0.02 *

NIPPV, non-invasive positive-pressure ventilation; NAVA, neurally adjusted ventilatory assist; CMV, conventional mechanical ventilation; HFOV, high-frequency oscillatory ventilation; HFJV, high-frequency jet ventilation; DOL, day of life; Wt, weight; MVET, mechanical ventilation with endotracheal tube. ^†^ Mean (SD); ^‡^ median (IQR); otherwise listed as N (%), * *p* = <0.05.

**Table 3:** As-treated respiratory and hospital length-of-stay outcomes.

	As Treated		*p*-Value
	NIPPV (N = 37)	NAVA (N = 20)	
**Pneumothorax**	7 (19%)	1 (5%)	0.24
**Extubation failure**	15 (41%)	7 (35%)	0.78
**Days extubated prior to first reintubation ^‡^**	2 (1, 6)	2 (1, 5)	0.83
**BPD mod/severe**	19 (51%)	4 (20%)	0.03 *
**O_2_ therapy (days) ^†^**	109 (54)	74 (26)	0.002 *
**O_2_ therapy at discharge**	16 (43%)	0 (0%)	0.0004 *
**Length of stay (days) ^‡^**	119 (86, 155)	98 (71, 118)	0.03 *
**PMA at discharge** ^‡^	42 (40, 47)	40 (37, 42)	0.02 *

NIPPV, non-invasive positive-pressure ventilation; NAVA, neurally adjusted ventilatory assist; BPD, bronchopulmonary dysplasia; PMA, postmenstrual age. ^†^ Mean (SD); ^‡^ median (IQR); otherwise listed as N (%), * *p* = <0.05.

**Table 4:** Intention to treat patient characteristics.

	Intention to Treat		*p*-Value
	NIPPV (N = 28)	NAVA (N = 29)	
**Birth weight, g ^†^**	774 (155)	772 (162)	0.95
**Gestational age, wks ^†^**	26 (1.7)	26 (1.2)	0.58
**C/S**	21 (75%)	23 (79%)	0.76
**Male**	14 (50%)	13 (45%)	0.79
**SGA**	5 (18%)	4 (14%)	0.73
**APGAR 1 min ≤ 7**	27 (96%)	28 (97%)	1
**AGPAR 5 min ≤ 7**	23 (82%)	23 (79%)	1
**APGAR 10 min ≤ 7**	15 (54%)	7 (24%)	0.24
**Prenatal steroids**	24 (86%)	27 (93%)	0.42
**PDA**	19 (68%)	22 (76%)	0.57
**Sepsis**	5 (18%)	2 (7%)	0.25
**IVH grade 3/4**	3 (11%)	3 (10%)	1

NIPPV, non-invasive positive-pressure ventilation; NAVA, neurally adjusted ventilatory assist; C/S, caesarean section; SGA, small for gestational age; PDA, patent ductus arteriosus; IVH, intraventricular hemorrhage. ^†^ Mean (SD); otherwise listed as N (%).

**Table 5:** Intention-to-treat respiratory characteristics.

	Intention to Treat		*p*-Value
	NIPPV (N = 28)	NAVA (N = 29)	
**Surfactant**	28 (100%)	29 (100%)	1
**Received > 1 dose surf**	8 (29%)	7 (24%)	0.77
**CMV days, pre-extubation ^‡^**	6 (1, 12)	4 (2, 11)	0.94
**HFOV days, pre-extubation ^‡^**	3 (0, 16)	3 (0, 13)	0.90
**HFJV days, pre-extubation ^‡^**	0 (0, 0)	0 (0, 0)	0.95
**DOL extubated ^‡^**	15 (1, 25)	8 (1, 37)	0.77
**Wt. at extubation g ^†^**	910 (167)	1013 (386)	0.20
**MVET total days ^‡^**	22 (8, 33)	20 (6, 42)	0.85
**Postnatal steroids**	8 (29%)	9 (31%)	1
**Diuretics**	4 (14%)	5 (17%)	1

NIPPV, non-invasive positive-pressure ventilation; NAVA, neurally adjusted ventilatory assist; CMV, conventional mechanical ventilation; HFOV, high-frequency oscillatory ventilation; HFJV, high-frequency jet ventilation; DOL, day of life; Wt, weight; MVET, mechanical ventilation with endotracheal tube. ^†^ Mean (SD); ^‡^ median (IQR); otherwise listed as N (%).

**Table 6:** Intention-to-treat respiratory and hospital length-of-stay outcomes.

	Intention to Treat		*p*-Value
	NIPPV (N = 28)	NAVA (N = 29)	
**Pneumothorax**	4 (14%)	4 (14%)	1
**Extubation failure**	12 (43%)	10 (34%)	0.59
**Days extubated prior to first reintubation ^‡^**	2 (1, 9)	1 (1, 4)	0.38
**BPD mod/severe**	14 (50%)	9 (31%)	0.18
**O_2_ therapy (days) ^†^**	96 (41)	97 (56)	0.98
**O_2_ therapy at discharge**	10 (36%)	6 (21%)	0.25
**Length of stay (days) ^‡^**	113 (83, 142)	109 (76, 140)	0.91
**PMA at discharge ^‡^**	42 (39, 46)	41 (38, 45)	0.71

NIPPV, non-invasive positive-pressure ventilation; NAVA, neurally adjusted ventilatory assist; BPD, bronchopulmonary dysplasia; PMA, postmenstrual age. ^†^ Mean (SD); ^‡^ median (IQR); otherwise listed as N (%).

## Data Availability

The original contributions presented in the study are included in the article, further inquiries can be directed to the corresponding author.
